# Regulatory Circuits That Enable Proliferation of the Fungus *Candida albicans* in a Mammalian Host

**DOI:** 10.1371/journal.ppat.1003780

**Published:** 2013-12-19

**Authors:** J. Christian Pérez, Alexander D. Johnson

**Affiliations:** Department of Microbiology and Immunology, University of California, San Francisco, San Francisco, California, United States of America; The University of North Carolina at Chapel Hill, United States of America

## A Rich Fungal Microbiome within Us

The human body harbors trillions of microorganisms—a rich and diverse microbiota that plays key roles in human health and disease. All three domains of life—eukaryotes, archaea, and bacteria—are represented in this large assembly of microbes. Among eukaryotes, fungi are particularly prominent residents of the human body. For example, 101 species belonging to 85 fungal genera have been found in the oral cavity of healthy people [1]. Similarly, >200 fungal species (half of them novel or unnamed) representing at least 50 genera have been found in the murine gut [2]. By comparison, there are ∼150 bacterial species (or phylotypes) inhabiting the mouths of healthy individuals [3] and ∼500–1000 bacterial species in their intestines [4], [5]. Although the methodologies differ, as do the classification schemes, it seems that the taxonomical diversity of the fungal component of the microbiota may not be so different from its bacterial counterpart, even though the latter has been much more heavily catalogued. With the exception of a handful of species, however, very little is known about the biology of the members of the human “mycobiome” (*i.e.*, fungal microbiome) and even less about the interactions that they establish with the human host.

In this review we focus on the most widely studied fungus that inhabits the human body, *Candida albicans*. Most, if not all, healthy adults carry this species asymptomatically in their gastrointestinal (GI) tract. Despite its status as a commensal microorganism, *C. albicans* is also a leading cause of mucosal disease in healthy hosts and systemic, life-threatening infections in individuals with debilitated immune systems, such as AIDS patients or people receiving chemotherapy. Not surprisingly, it is this “virulent” or “pathogenic” facet of *C. albicans* that has traditionally received the most attention from researchers. More recently, however, several laboratories have begun to explore the tactics that the fungus uses to proliferate in its more common niche, the mammalian gut. These studies have uncovered unexpected connections between the commensal and pathogenic lifestyles. We highlight some of these findings here.

## Regulation of Nutrient Acquisition Is Pivotal for Colonizing the Mammalian Gut

The gut is a crowded environment. In the colon alone, *C. albicans* cohabits with ∼10^11^ microbial cells per milliliter of intestinal content [6]. Food resources may be plentiful but competition is obviously high (reviewed in [7]). The ability of *C. albicans* to colonize and proliferate in the GI tract has been studied in mice receiving antibiotics orally; while these animals are not natural hosts of *C. albicans*, they likely serve as reasonable proxies. Because transcription regulators are central elements within the gene network of any organism, the use of them as entry points to the dissection of this trait has proved an effective strategy. A recent screen carried with *C. albicans* in a mouse model of GI tract colonization [8] evaluated 77 transcription regulator mutants for their ability to endure in the intestine for several weeks after oral inoculation. This group of mutants was chosen because none of them showed overt defects under a wide variety of conditions *in vitro* [9], hence they would be candidates for regulating circuits needed specifically *in vivo*. The screen identified six transcription regulators required for *C. albicans* to colonize the murine gut, four of which (*RTG1*, *RTG3*, *TYE7*, and *LYS144*) controlled the expression of genes responsible for the acquisition and metabolism of nutrients, particularly carbon and nitrogen sources. A significant fraction of the target genes of these regulators (identified by full-genome chromatin immunoprecipitation experiments) are indeed upregulated in *C. albicans* cells growing in the mouse intestine [10]. It is noteworthy that none of the four regulators is required for the species to grow under standard laboratory conditions [9], which reinforces the notion that significant resources are devoted to simply obtain food in the GI tract. It is plausible that these stringent conditions are set, at least in part, by the presence of a competing gut microbiota; consistent with this idea, genes that enable murine gut colonization by intestinal microbes such as *Citrobacter rodentium*—a natural mouse pathogen—are not required to colonize the intestines of germ-free mice [11].

## Managing Iron Toxicity in the Mammalian Gut

Iron is an essential nutrient for microorganisms but at high levels it can become extremely toxic. The abundance and availability of iron vary greatly in different locales of the human body: relatively high levels (∼10^−4^ M) are found in the GI tract as the majority of dietary iron is not absorbed [12], whereas the concentration in the bloodstream is orders of magnitude lower (∼10^−24^ M free Fe^3+^) [13]. *C. albicans*, an organism that typically inhabits the gut but that can also cross into the bloodstream, harbors a regulatory circuit composed of three transcription regulators (*SFU1*, *SEF1*, and *HAP43*) that controls the expression of iron uptake genes and iron utilization genes [14]. *SFU1* is required to persist in the GI tract [14] and for resistance to iron toxicity *in vitro* [9], suggesting that the primary role of this circuit in the gut is to protect *C. albicans* from iron's noxious effects. *SEF1*, on the other hand, is required not only for the fungus to endure in the intestine but also for full virulence after bloodstream infection [14]. Thus, *C. albicans* uses a common circuitry to balance its need to uptake iron in one niche (*i.e.*, in the bloodstream) and protect itself from its toxic effects in other locale (*i.e.*, the GI tract).

In addition to circuits that govern carbon, nitrogen, and iron acquisition, gene products that function as adherence molecules [15] and in the detoxification of reactive oxygen species [16] have also been shown to play roles in gut colonization. In fact, *ECE1* and *SOD5*, which influence adhesion and protection against reactive oxygen species, respectively, are among the most highly upregulated genes when *C. albicans* is growing in the GI tract compared to standard laboratory conditions [15], [16]. Host cells such as macrophages and neutrophils produce reactive oxygen species, raising the possibility that *SOD5*'s role may be to defend the fungus from these molecules [16] during colonization of the GI tract. Other transcription regulators reported to play roles in gut colonization have been shown to be rather pleiotropic (*e.g.*, *EFG1* [17], *CPH2* [10], and *EFH1* [15]).

## A Molecular Basis for the Interplay of Commensalism and Pathogenicity

Many systemic, life-threatening infections in humans are caused by the very same bacteria or fungi that compose their microbiota. That is, these opportunistic pathogens reside in the host as harmless commensals (typically on mucosal surfaces) but can cross the host's protective barriers and colonize internal organs causing serious disease. The GI tract, for instance, appears to be the ultimate source of the majority of deep-seated *C. albicans* infections [18], [19]. Do entirely different gene sets account for the seemingly disparate behaviors of *C. albicans* as commensal versus pathogen?

Observing phenotypes on a gene-by-gene basis provides a mixed answer. On the one hand, transcription regulators such as *EFG1* [16], [17], *SEF1* [14], *RTG1*, *RTG3*, and *HMS1* [8] are necessary for full virulence in mouse models of systemic infection (*i.e.*, as a pathogen) as well as for GI tract colonization (*i.e.*, as a commensal). On the other hand, mutant strains lacking the regulators *TEC1* or *LYS14* show reduced virulence in models of systemic infection but display no obvious defect in colonizing the murine GI tract [8], [10], [20]. The converse is true for *C. albicans* strains lacking *TYE7* or *SFU1* or ectopically expressing *EFH1* [8], [14], [15]. However, genes do not function in isolation, and a clearer picture emerges by considering the existing links among some of these regulators.

**Figure 1:**
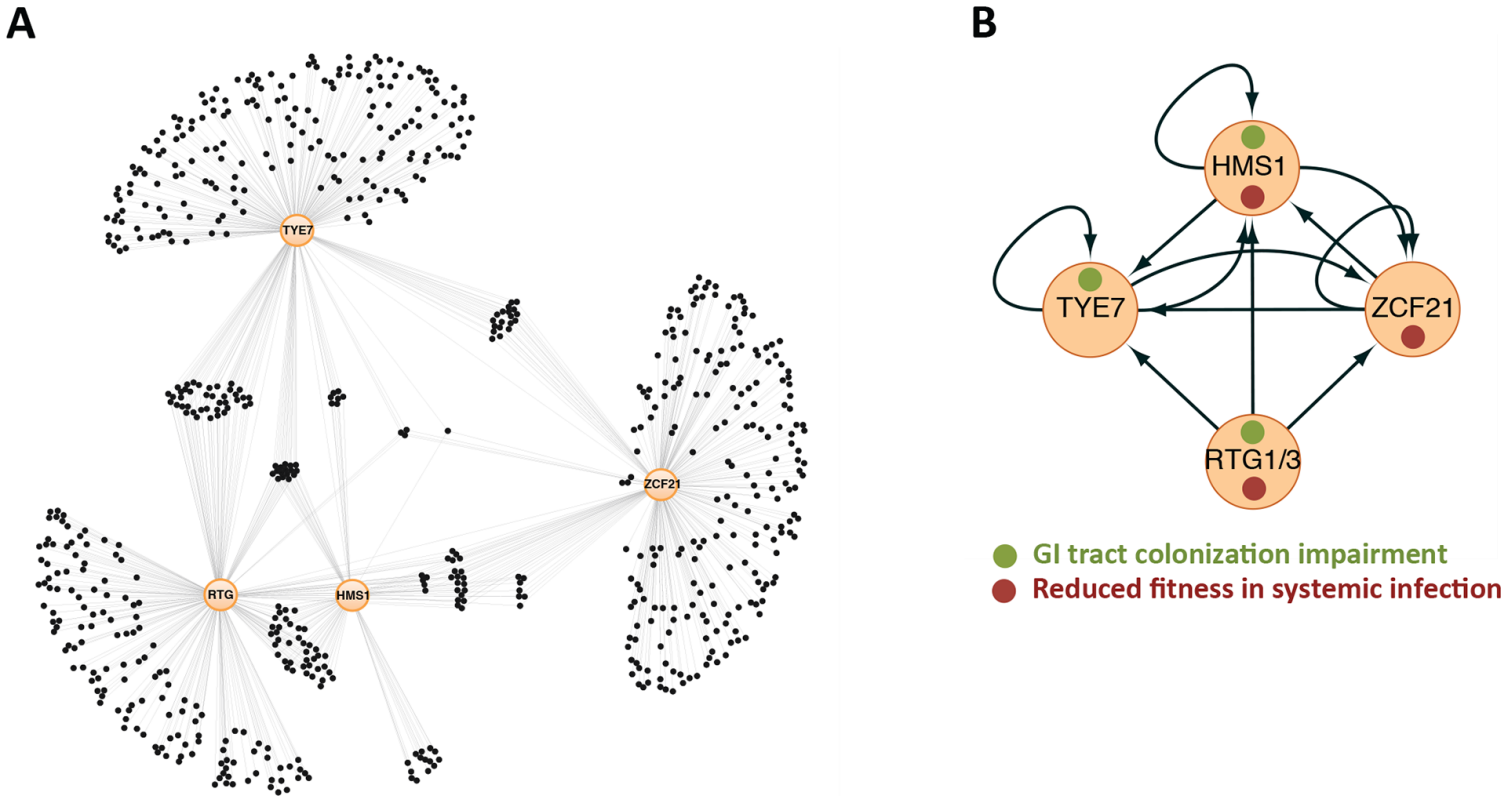
Gene regulatory network directing *C. albicans* proliferation in a mammalian host. (A) Gene network composed of the transcription regulators *RTG1/3*, *HMS1*, *ZCF21*, and *TYE7* (orange circles) and their target genes (black circles) as determined by full-genome chromatin immunoprecipitation. Thin lines indicate binding of the specified regulator to a target gene. About a quarter of the genes in the network (those in the middle) are targets shared by two or more regulators. (B) Relationships among the “master” regulators at the core of the network. Arrows represent protein-DNA interactions. Notice that the circuit displays multiple autoregulatory, feed-forward, and feedback loops. Adapted from [8].

Five transcription regulators that displayed a significant phenotype “exclusively” in association with the host [8] form a tightly knit circuit (Figure 1). In this circuit, the regulators are connected with one another irrespective of whether they are required for GI-tract colonization, systemic infection, or both. This observation implies that significant portions of the “pathogenic” and “commensal” lifestyles are coupled and controlled by the same core circuitry. Thus, from a gene network perspective, commensalism and pathogenicity do not appear to represent fully independent traits but rather are intertwined. The close links between these two traits may reflect the natural history of *C. albicans*: its association with mammals is ancient [21] and the selection pressure on the fungus has likely been as a commensal organism. Thus, it is plausible that the functions employed by *C. albicans* to spread from the human gut into systemic infections rely on the same regulatory circuitry that evolved to enable growth in the host as a commensal organism. Consistent with this idea, some impairment in the host immune system is typically required for *C. albicans* to be fully pathogenic.

## Commensalism/Pathogenicity Transcriptional Circuit in *C. albicans* Has Structure in Common with Circuits Underlying Cell Differentiation

It has been shown that at the heart of the ability of *C. albicans* to proliferate in the mammalian host lies a highly interconnected transcriptional circuit composed of five transcription regulators (Figure 1). The distinctive feature of this circuit is the existence of multiple connections among all its components; that is the “master” regulators control one another (in addition to their target genes), and the target genes are typically bound by multiple master regulators. Thus, the circuit has many feed-forward and feedback loops. By contrast, many “textbook” genetic circuits—those that most scientists have grown used to—consist of simple, unidirectional relationships among their components, making their behavior more predictable. Because of the distinctly interwoven appearance of the *C. albicans* circuit, it is difficult to predict its behavior without extensive knowledge of the parameters (*e.g.*, concentration of proteins, affinity constants, and the like). It is noteworthy that the overall topology resembles other circuits known to direct well-established cell differentiation processes such as white-opaque switching [22] and biofilm development [23] in *C. albicans*, filamentation in *S. cerevisiae* [24], and embryonic development in metazoans (*e.g.*, see [25]). The fact that disparate functions in divergent organisms rely on a similar network architecture suggests that these transcriptional circuits may share common, underlying features. It is possible, for example, that the network structure enables the integration of multiple internal and external cues to then specify the precise pattern of target gene expression.

## Conclusion

The study of opportunistic pathogens such as the fungus *C. albicans*, which can proliferate in disparate niches of the host either as a commensal or a pathogenic organism, allows us to genetically dissect the features of these two ways of life as well as to reveal the links between them. The results thus far suggest that there may be no clear distinction between the genetic circuitries employed during “harmless” proliferation in the gut or “disease-causing” growth after bloodstream infection. Rather, a single highly interconnected transcriptional circuit (one whose structure resembles others that orchestrate cell differentiation) may govern proliferation in all niches of the mammalian host.

## References

[1] Ghannoum MA, Jurevic RJ, Mukherjee PK, Cui F, Sikaroodi M, et al (2010) Characterization of the oral fungal microbiome (mycobiome) in healthy individuals. PLoS Pathog 6: e1000713 doi:10.1371/journal.ppat.1000713 20072605 10.1371/journal.ppat.1000713 PMC2795202

[2] Iliev ID, Funari VA, Taylor KD, Nguyen Q, Reyes CN, et al (2012) Interactions between commensal fungi and the C-type lectin receptor Dectin-1 influence colitis. Science 336: 1314–1317. 22674328 10.1126/science.1221789 PMC3432565

[3] Aas JA, Paster BJ, Stokes LN, Olsen I, Dewhirst FE (2005) Defining the normal bacterial flora of the oral cavity. J Clin Microbiol 43: 5721–5732. 16272510 10.1128/JCM.43.11.5721-5732.2005 PMC1287824

[4] Human Microbiome Project Consortium (2012) Structure, function and diversity of the healthy human microbiome. Nature 486: 207–214. 22699609 10.1038/nature11234 PMC3564958

[5] Qin J, Li R, Raes J, Arumugam M, Burgdorf KS, et al (2010) A human gut microbial gene catalogue established by metagenomic sequencing. Nature 464: 59–65. 20203603 10.1038/nature08821 PMC3779803

[6] Walter J, Ley R (2011) The human gut microbiome: ecology and recent evolutionary changes. Ann Rev Microbiol 65: 411–429. 21682646 10.1146/annurev-micro-090110-102830

[7] Fischbach MA, Sonnenburg JL (2011) Eating for two: how metabolism establishes interspecies interactions in the gut. Cell Host Microbe 10: 336–347. 22018234 10.1016/j.chom.2011.10.002 PMC3225337

[8] Pérez JC, Kumamoto CA, Johnson AD (2013) *Candida albicans* commensalism and pathogenicity are intertwined traits directed by a tightly knit transcriptional regulatory circuit. PLoS Biol 11: e1001510 doi:10.1371/journal.pbio.1001510 23526879 10.1371/journal.pbio.1001510 PMC3601966

[9] Homann OR, Dea J, Noble SM, Johnson AD (2009) A phenotypic profile of the *Candida albicans* regulatory network. PLoS Genet 5: e1000783 doi:10.1371/journal.pgen.1000783 20041210 10.1371/journal.pgen.1000783 PMC2790342

[10] Rosenbach A, Dignard D, Pierce JV, Whiteway M, Kumamoto CA (2010) Adaptations of *Candida albicans* for growth in the mammalian intestinal tract. Eukaryot Cell 9: 1075–1086. 20435697 10.1128/EC.00034-10 PMC2901676

[11] Kamada N, Kim YG, Sham HP, Vallance BA, Puente JL, et al (2012) Regulated virulence controls the ability of a pathogen to compete with the gut microbiota. Science 336: 1325–1329. 22582016 10.1126/science.1222195 PMC3439148

[12] Miret S, Simpson RJ, McKie AT (2003) Physiology and molecular biology of dietary iron absorption. Annu Rev Nutr 23: 283–301. 12626689 10.1146/annurev.nutr.23.011702.073139

[13] Martin RB, Savory J, Brown S, Bertholf RL, Wills MR (1987) Transferrin binding of Al^3+^ and Fe^3+^ . Clin Chem 33: 405–407. 3815806

[14] Chen C, Pande K, French SD, Tuch BB, Noble SM (2011) An iron homeostasis regulatory circuit with reciprocal roles in *Candida albicans* commensalism and pathogenesis. Cell Host Microbe 10: 118–135. 21843869 10.1016/j.chom.2011.07.005 PMC3165008

[15] White SJ, Rosenbach A, Lephart P, Nguyen D, Benjamin A, et al (2007) Self-regulation of *Candida albicans* population size during GI colonization. PLoS Pathog 3: e184 doi:10.1371/journal.ppat.0030184 18069889 10.1371/journal.ppat.0030184 PMC2134954

[16] Pierce JV, Dignard D, Whiteway M, Kumamoto CA (2013) Normal adaptation of *Candida albicans* to the murine gastrointestinal tract requires Efg1p-dependent regulation of metabolic and host defense genes. Eukaryot Cell 12: 37–49. 23125349 10.1128/EC.00236-12 PMC3535844

[17] Pierce JV, Kumamoto CA (2012) Variation in *Candida albicans EFG1* expression enables host-dependent changes in colonizing fungal populations. mBio 3: e00117–00112. 22829676 10.1128/mBio.00117-12 PMC3413400

[18] Cole GT, Halawa AA, Anaissie EJ (1996) The role of the gastrointestinal tract in hematogenous candidiasis: from the laboratory to the bedside. Clin Infect Dis 22 Suppl 2: S73–88. 8722833 10.1093/clinids/22.supplement_2.s73

[19] Miranda LN, van der Heijden IM, Costa SF, Sousa AP, Sienra RA, et al (2009) *Candida* colonisation as a source for candidaemia. J Hosp Infect 72: 9–16. 19303662 10.1016/j.jhin.2009.02.009

[20] Schweizer A, Rupp S, Taylor BN, Rollinghoff M, Schroppel K (2000) The TEA/ATTS transcription factor CaTec1p regulates hyphal development and virulence in *Candida albicans* . Mol Microbiol 38: 435–445. 11069668 10.1046/j.1365-2958.2000.02132.x

[21] Odds FC (1987) *Candida* infections: an overview. CRC Cr Rev Microbiol 15: 1–5. 10.3109/10408418709104444 3319417

[22] Zordan RE, Miller MG, Galgoczy DJ, Tuch BB, Johnson AD (2007) Interlocking transcriptional feedback loops control white-opaque switching in *Candida albicans* . PLoS Biol 5: e256 doi:10.1371/journal.pbio.0050256 17880264 10.1371/journal.pbio.0050256 PMC1976629

[23] Nobile CJ, Fox EP, Nett JE, Sorrells TR, Mitrovich QM, et al (2012) A recently evolved transcriptional network controls biofilm development in *Candida albicans* . Cell 148: 126–138. 22265407 10.1016/j.cell.2011.10.048 PMC3266547

[24] Borneman AR, Leigh-Bell JA, Yu H, Bertone P, Gerstein M, et al (2006) Target hub proteins serve as master regulators of development in yeast. Gene Dev 20: 435–448. 16449570 10.1101/gad.1389306 PMC1369046

[25] Neph S, Stergachis AB, Reynolds A, Sandstrom R, Borenstein E, et al (2012) Circuitry and dynamics of human transcription factor regulatory networks. Cell 150: 1274–1286. 22959076 10.1016/j.cell.2012.04.040 PMC3679407

